# Approaching Autism Diagnosis and Care Through the Lens of Gender Diversity

**DOI:** 10.1177/2329048X231219201

**Published:** 2023-12-17

**Authors:** Margaret Goss, Carolyn K. Huynh, Matthew Taing, Audrey C. Brumback

**Affiliations:** 1 UT Health Austin Pediatric Neurosciences at Dell Children's Medical Center, Austin, TX, USA; 2 377659 Dell Medical School, University of Texas at Austin, Austin, TX, USA; 3 Departments of Neurology and Pediatrics, Dell Medical School, Austin, TX, USA; 4 Center for Learning and Memory, 377659 University of Texas at Austin, Austin, TX, USA

## Introduction

Autism is a neurodevelopmental condition that is particularly prevalent in patients with neurological disorders.^1–3^ Gender dysphoria and autism often co-occur,^4^ and can make the diagnosis of autism more challenging for clinicians. Here, we discuss the case of a transgender male whose autism was evident early in life but was not diagnosed as autism until adolescence. We provide recommendations for general neurologists regarding the intersection of gender diversity and neurodiversity: 1) Autism is under-diagnosed in girls, so consider autism as a possible diagnosis in older cisgender females and transgender males presenting with symptoms of ADHD, anxiety, depression, obsessive-compulsive disorders, personality disorder, or academic difficulties; 2) Autism and transgender often co-occur, so consider screening for autism in transgender patients. When evaluating transgender males, be aware that autism may not have been recognized earlier in life when the person presented as female. 3) Consider having conversations about gender identity as part of routine clinical care for patients with autism; and 4) Screen for and treat anxiety and depression in autistic transgender patients.

## Case

John is a 13-year-old boy who was assigned female at birth. He was born full-term to a 38-year-old G1P1 mother and 45-year-old father, after an unremarkable pregnancy. Early developmental milestones were met on time, including first words around 12 months and walking around 13 months. Since the age of 11, he made it known to his family that he identifies as male and changed his name to John. At age 13, John presented to our pediatric neurology clinic for evaluation because he felt “neurodivergent”. When we asked John during our initial visit how he viewed his own gender, he responded, “I don’t really like to talk about it.”

From an early age, John displayed atypical social communication. Throughout preschool and elementary school, he enjoyed being around other children but did not play with them. John's mother described his social interactions as “parallel play”. In first grade, John was diagnosed with dyslexia. Despite struggling with dyslexia, he loved to read and was content reading alone at recess. John's teacher reported that he was disconnected from his surroundings and sometimes alarmingly alone, had difficulty making friends, and was unable to join group activities. John recalls that he did not know what to say when interacting with peers.

Adults in John's life describe him as creative and artistic. John's therapist reported that he was a very intelligent, verbal, and highly talented person with an incredible imagination. Yet, John struggled to express his emotions and would shut down around other people. John's mother said that it was difficult to tell what he was feeling based on his facial expressions. John tended to not focus on other people's feelings and struggled to identify his own emotions (alexithymia).

John was often deeply engrossed in his interests such as Littlest Pet Shop figurines. John's therapist observed that John could be so engaged in his interests that it was hard for him to do other things. John also adhered to rituals, such as unwrapping cupcakes in a very specific way. He received occupational therapy for repetitively biting his clothing and as a teenager started wearing a chewable necklace to redirect this behavior. He did not have gross motor stereotypies. Regarding sensory processing, John reported that there was sometimes too much sensory stimulation. For example, he had a strong aversion to many odors and felt distress in loud environments.

Based on history and observation, John met DSM-5 criteria for autism spectrum disorder (ASD).

## Discussion

### Autism and Gender Dysphoria co-Occur

Children with ASD are more likely than typically-developing children to be diagnosed with gender dysphoria (GD).^5,6^ Conversely, individuals with GD have a higher incidence of autism than the general population.^7^ The co-occurrence of ASD and GD may have an impact on the recognition of ASD. Children typically exhibit autism symptoms by 12 to 18 months,^8–10^ and are diagnosed on average by age 4 years.^11^ For children with both ASD and GD, however, autism can go undiagnosed for several years. In one study, children with GD were recognized as having ASD at age 11.3 years versus at 6.5 years for children without GD.^5^

### Societal Gender Stereotypes Play a Role in the Diagnostic Process

Autism is more frequently diagnosed in males than females.^12–14^ It is more common for girls with autism to first be diagnosed with intellectual disability, ADHD, general developmental delay, or epilepsy rather than ASD.^15^ Girls with autism then experience diagnostic overshadowing (incorrectly attributing symptoms to a co-occurring disorder), where their ASD symptoms are attributed to the diagnoses they already carry such as ADHD or social anxiety. This tendency to diagnose ASD more readily in boys is also present in patients with GD; 80% of patients with GD and ASD were assigned male at birth.^5,6^

One major contribution to the disparity between male and female diagnosis rates may be that autism often presents differently in girls and boys.^16–19^ Female autism phenotype characteristics include awareness of the need for social interaction, greater likelihood to “camouflage” or “mask” difficulties within social interactions (especially by imitating others in social interactions - “social echolalia”), having one or a few close friendships, better language development, better imagination, restricted interests involving people and animals (as opposed to objects), fewer gross motor stereotypies, the tendency towards perfectionism and controlling behaviors, and the tendency for eating disorders.^20–24^ The overlap of female autism phenotypes with societal expectations of girls may contribute to why females with autism are underdiagnosed compared to their male counterparts.^12,25^

As autism awareness increases, patients like John are now presenting in adolescence for evaluation of previously undiagnosed autism.^26^ We suspect that John's autism was not recognized earlier in life because he was assigned female at birth and his symptoms were consistent with a female autism phenotype.

### Do Autism Symptoms Track with Sex Assigned at Birth or with Gender Identity?

In a person who is transgender, would we expect their symptoms to be expressed per the sex assigned at birth as they are in John, or, by contrast, in concordance with their gender identity? The answer to this question is currently unknown, but autism symptoms may be detected prior to the establishment of gender identity. Children typically start exhibiting symptoms of autism by 12 to 18 months.^27^ Awareness of sex assigned at birth also can begin by age 2 years.^28^ Regarding gender identify, between ages 3 and 5 years, many children believe that their gender will remain static through adulthood.^29^ Some transgender adults report experiencing gender dysphoria for the first time around that age as well (3-7 years old),^30^ but some children like our patient may first experience gender dysphoria later in life. In summary, based on our current understanding of developmental timing, it is difficult to predict if autism symptoms would tend to be more congruent with sex assigned at birth or with gender identity.

### Recommendations for Evaluating and Treating Autism in Transgender Populations

#### Consider Autism as a Possible Diagnosis in Cis or Trans Females and Trans Males Carrying Diagnoses of ADHD, Anxiety, Depression, Obsessive-Compulsive Disorder, a Personality Disorder, or Academic Difficulties

Autism may not be recognized early in life, even by experienced clinicians, for several reasons. Girls with normal intelligence often “camouflage” or adhere to social norms^31–33^ so they can fly under the radar, so to speak. Differences between autistic and non-autistic girls may not become apparent until adolescence, whereas for boys these differences are often noted in elementary school.^34^ Simply the belief that autism is more common in males may contribute to a self-fulfilling prophecy. The clinician evaluating an older child or teenager may not even include autism on their differential diagnosis because they believe autism would have been recognized at an earlier age. Instead of their autism being recognized as the explanation for their complex clinical phenotype, women with autism often receive dual diagnoses such as ADHD and anxiety. This diagnostic overshadowing can prematurely end the diagnostic journey and decrease the likelihood future clinicians will consider autism on the differential diagnosis.

#### Consider Evaluating for Autism in Transgender Patients or Patients with Gender Dysphoria Presenting with Neuropsychiatric Symptoms, as the Diagnosis of Autism may Have Been Missed Earlier in Life When the Person Presented as Female

Another form of diagnostic overshadowing can occur when the symptoms of autism are incorrectly attributed to the person's gender dysphoria. On the opposite hand, transgender patients may experience others doubting their gender identity because their gender dysphoria is incorrectly interpreted as a symptom of autism.^35^ Instead, recognize that autism and gender dysphoria often co-occur.^36^ Importantly, the co-occurrence of autism and gender dysphoria does not necessarily imply a causal relationship or directional influence between the two. Of note, transgender adolescents newly diagnosed with autism may find the autism diagnosis more difficult to process than their gender differences.^37^

#### Be Cognizant of and Prepared to Have Conversations About Gender Identity as Part of Routine Care of Patients with Autism

Discussion of the theories exploring the overlap of neurodiversity (e.g., autism) and gender diversity are beyond the scope of this case report.^38–40^ It is important to note that one common symptom of autism, alexithymia (difficulty with identifying, expressing, and/or describing one's feelings), may play a role in gender identity differences in people with ASD.^41,42^ Because of alexithymia, autistic individuals may have difficulties discussing gender discernment and may lack the ability to advocate for their gender needs.^37^

As of 2020, one in 36 children has been identified with ASD.^14^ As autistic children become autistic teens and adults, clinicians need to be prepared to have conversations about gender identity as part of routine care.^4^ If gender concerns arise, not having these conversations can worsen gender dysphoria and promote stigmatization.^43^ If clinicians do not feel comfortable engaging in conversations about gender identity, it may be appropriate to refer patients to consulting providers to address these concerns.^44^

#### Screen for and Treat Anxiety and Depression in Autistic Transgender Patients

Autistic transgender individuals have high rates of depression and anxiety.^45^ Because of diagnostic overshadowing, mood symptoms may be incorrectly ascribed to gender dysphoria and vice versa. When caring for an autistic transgender patient who is experiencing mood disorder symptoms, be aware that the mood symptoms might not entirely be attributable to gender dysphoria. As co-occurring conditions, treatment of one may not ameliorate the other. We therefore recommend treating gender dysphoria with appropriate gender-affirming care and simultaneously treating anxiety and depression with the appropriate mood-disorder treatments. Delaying gender-affirming care to prioritize the treatment of a co-occurring mood disorder or vice versa may adversely affect the patient’s overall mental health.

## Conclusion

Gender dysphoria and autism co-occur more commonly than neurologists may be aware of. Accurately diagnosing patients with gender dysphoria and/or autism opens the door for the appropriate physical and mental care that patients need and deserve. In the case of our transgender patient John, his autism was not diagnosed until he was 13 years old, despite his showing signs of autism as a young child. Societal gender roles and gender differences in autism phenotypes may have contributed to John's and other patients’ late diagnoses. Identifying autism in gender-diverse patients can help end the diagnostic odyssey and provide a roadmap for future treatments.
